# Incorporating Cellular Stochasticity in Solid–Fluid Mixture Biofilm Models

**DOI:** 10.3390/e22020188

**Published:** 2020-02-06

**Authors:** Ana Carpio, Elena Cebrián

**Affiliations:** 1 Departamento de Matemática Aplicada, Universidad Complutense, 28040 Madrid, Spain; 2 Departamento de Matemáticas y Computación, Universidad de Burgos, 09001 Burgos, Spain; elenac@ubu.es

**Keywords:** biofilm, cellular activity, solid–fluid mixture, thin film, Von Karman plate, dynamic energy budget, osmotic spread, wrinkle formation, cell differentiation

## Abstract

The dynamics of cellular aggregates is driven by the interplay of mechanochemical processes and cellular activity. Although deterministic models may capture mechanical features, local chemical fluctuations trigger random cell responses, which determine the overall evolution. Incorporating stochastic cellular behavior in macroscopic models of biological media is a challenging task. Herein, we propose hybrid models for bacterial biofilm growth, which couple a two phase solid/fluid mixture description of mechanical and chemical fields with a dynamic energy budget-based cellular automata treatment of bacterial activity. Thin film and plate approximations for the relevant interfaces allow us to obtain numerical solutions exhibiting behaviors observed in experiments, such as accelerated spread due to water intake from the environment, wrinkle formation, undulated contour development, and the appearance of inhomogeneous distributions of differentiated bacteria performing varied tasks.

## 1. Introduction

Bacterial biofilms provide basic model environments for analyzing the interaction between mechanical and cellular aspects of three-dimensional self-organization during development. Biofilms are formed when bacteria encase themselves in a hydrated layer of self-produced extracellular matrix (ECM) made of exopolymeric substances (EPS) [1]. This habitat confers them enhanced resistance to disinfectants, antibiotics, flows, and other mechanical or chemical agents [2].

Research on modeling biofilms has increased steadily during the past few decades resulting in the understanding of a number of features. Continuous models for uniform cell distributions are useful in basic culture systems [3]. Individual based models [4,5] and cellular automata [6] may capture variable thickness, density, and structure. However, current models focus more on deterministic mass transfer and extracellular structure, than in random cell processes. Interest on fluctuations in intracellular concentrations, for instance, has arisen due to their significance in phenotypic variability as well as in gene regulation and stochasticity of gene expression [7,8], with consequences for development and drug resistance [9].

**Figure 1:**
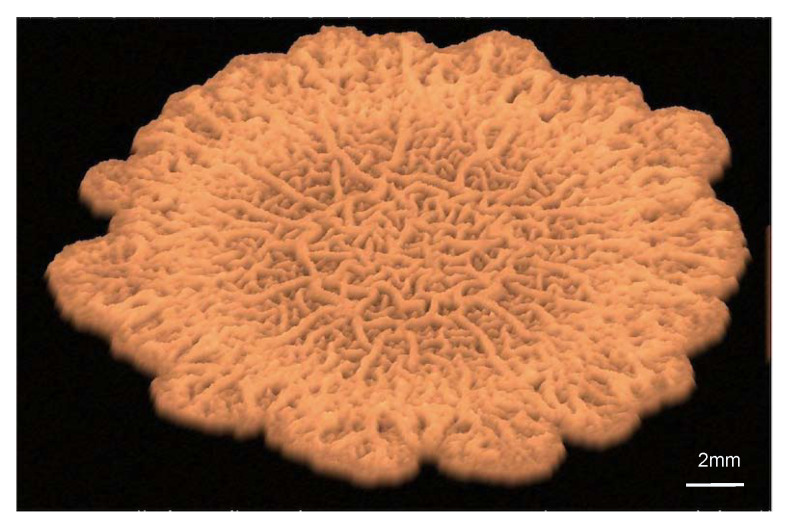
Virtual visualization of a biofilm spreading on agar.

Recent experiments with *Bacillus subtilus* biofilms on agar provide a case study in which we can test models incorporating new aspects. Once bacteria adhere to a surface, they differentiate in response to local fluctuations created by growth, death, and division processes, to variations in the concentrations of nutrients, waste, and autoinducers, to cell–cell communication [10]. Some of them become producers of exopolymeric substances (EPS) and form the extracellular matrix (ECM). EPS production increases the osmotic pressure in the biofilm, driving water from the agar substrate and accelerating spread [11]. In addition, the matrix confers the biofilm elastic properties. Wrinkles develop as the result of localized death in regions of high cell density and compression caused by division and growth [12]. As the biofilm expands, complex wrinkled patterns develop, see Figure 1. This phenomenon is linked to gradients created by heterogeneous cellular activity and water migration [13]. Eventually, the wrinkles form a network of channels transporting water, nutrients, and waste to sustain it [14,15]. Biofilm spread due to osmosis can be accounted for by two-phase flow models and thin film approximations [11]. Instead, wrinkle formation has been reproduced by means of Von Kármán-type theories [13,16]. Delamination and folding processes are further analyzed in [17] by means of neo-Hookean models. In [18], a poroelastic approach provides a unified description of liquid transport and elastic deformations in the biofilm. To incorporate fluctuations in a more natural way, here we propose a mixture model allowing to distinguish the different phenotypes forming the film.

Biofilm structure is greatly influenced by environmental conditions. When they grow in flows, we find bacteria immersed in large lumps of polymer, typically forming fingers and streamers [19] in the surrounding current. In contrast, biofilms spreading on air–agar interfaces contain small volume fractions of extracellular matrix [11], producing wrinkled shapes with internal water flow. This motivates different treatments of the extracellular matrix, see [20,21] for biofilms in flows and [4,5,11,22] for biofilms on interfaces with air or tissues, for instance. In the latter case, when internal fluid flow is taken into account, the small fraction of matrix is usually merged in one biomass phase with the cells [11,22]. Some experimental studies suggest a viscoelastic rheology for biofilms [23,24]. The analysis of the mixture and poroelastic models we consider shows that, depending on the volume fractions of solid biomass and fluid, the viscosity of the fluid, the Lamé constants of the solid, the densities, and hydraulic permeability of the fluid/solid system, the characteristic time for variations in the displacement of the solid, and the characteristic length of the network in the macroscopic scale, the resulting mixture can be considered as monophasic elastic, monophasic viscoelastic, or truly biphasic mixture/poroelastic [25,26].

The paper is organized as follows. Section 2 introduces the solid–fluid mixture model. Section 3 discusses ways to incorporate details of cell behavior. We present a cellular automata approach based on dynamic energy budget descriptions of bacterial metabolism. With the aid of asymptotic analysis [11,27], we construct numerical solutions displaying behaviors consistent with experimental observations. Finally, Section 4 discusses our results, the advantages, and limitations of our approach, as well as future perspectives and possible improvements.

## 2. Solid-Fluid Mixture Model of a Biofilm Spreading on an Agar/Air Interface

**Figure 2:**
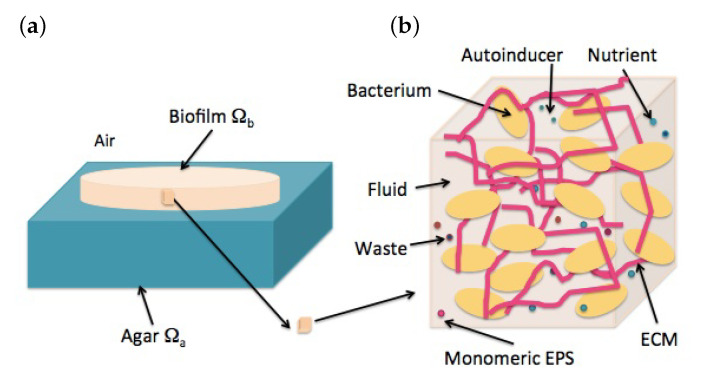
Schematic structure of a biofilm: (**a**) View of the macroscopic configuration: a biofilm on an agar–air interface. (**b**) Microstructure formed by biomass (polymeric mesh and cells) and fluid containing dissolved substances (nutrients, waste, and autoinducers).

In this section, we adapt bicomponent mixture models of swelling tissues [22,28] to describe the spread of biofilms of air–agar interfaces, including biomass variations. We consider the biofilm as a bicomponent mixture of incompressible solid matrix (bacterial cells and polymers) and interstitial fluid carrying nutrients, waste, and autoinducers, see Figure 2. The biofilm occupies a region $\Omega_{b}\left(t\right),$ placed over an agar substratum $\Omega_{a}\left(t\right)$ and in contact with air. Large immobilized solutes are considered part of the extracellular matrix (ECM). Small molecules diffusing rapidly are considered part of the fluid.

### 2.1. Mass Balance

Under the equipresence hypothesis of mixtures, each point x in the biofilm can be occupied simultaneously by both phases. In addition, we assume that no air bubbles or voids form inside the biofilm. If $\phi_{s}\left(x,t\right)$ denotes the volume fraction of solid and $\phi_{f}\left(x,t\right)$ the volume fraction of fluid, then $\phi_{s}+\phi_{f}=1.$ The standard densities of biological tissues and agar are similar to the density of water $\rho_{f}=10^{3}\;\mathrm{kg}/m^{3}$ (typical relative differences of order $10^{-2}$). Thus, we will take the densities of all constituents and the mixture to be constant and equal to that of water [11]: $\rho_{f}=\rho_{s}=\rho =\rho_{w}.$ Then, the balance laws for the fractions of solid biomass $\phi_{s}$ and fluid $\phi_{f}$ are [11,22]

(1) $$\begin{array}{c} \frac{\partial \phi_{s}}{\partial t}+\mathrm{div}\left(\phi_{s}v_{s}\right)=r_{s}\left(\phi_{s},c_{n}\right),\;\frac{\partial \phi_{f}}{\partial t}+\mathrm{div}\left(\phi_{f}v_{f}\right)=-r_{s}\left(\phi_{s},c_{n}\right), \end{array}$$

where $r_{s}\left(\phi_{s},c_{n}\right)$ represents biomass production due to nutrient consumption, whereas $v_{s}$ and $v_{f}$ stand for the velocities of the solid and the fluid components, respectively. Biomass production can be accounted for through a Monod law $r_{s}\left(\phi_{s},c_{n}\right)=k_{s}\frac{c_{n}}{c_{n}+K_{n}}\phi_{s}=\frac{1+\alpha_{m}}{\tau }\frac{c_{n}}{c_{n}+K_{n}}\phi_{s}=g\left(c_{n}\right)\phi_{s},$ where $c_{n}$ is the nutrient concentration, $K_{n}$ is a constant that marks the onset of starvation, and $k_{s}$ the uptake rate, which can be approximated by $\frac{1+\alpha_{m}}{\tau }$, τ being the doubling time for the specific bacteria and $\alpha_{m}$ a factor representing polymeric matrix production [11].

Adding up Equation (1), we find a conservation law for the growing mixture:

(2) $$\begin{array}{c} 0=\mathrm{div}\left(\phi_{s}v_{s}+\phi_{f}v_{f}\right)=\mathrm{div}\left(v\right)=\mathrm{div}\left(v_{s}+q\right), \end{array}$$

where $v=\phi_{s}v_{s}+\phi_{f}v_{f}$ is the composite velocity of the mixture and

(3) $$\begin{array}{c} q=\phi_{f}\left(v_{f}-v_{s}\right)=\phi_{f}w \end{array}$$

is the filtration flux, w being the relative velocity.

### 2.2. Driving Forces

Forces inducing motion are of a different nature: inner stresses, inertial forces, interactive forces, and external body forces. We discuss here the constitutive relations and fluxes for incompressible solid–fluid mixtures in the case of infinitesimal deformations and under isothermal conditions [22].

#### 2.2.1. Stresses in the Solid and the Fluid

When a large number of small pores are present in the biofilm, the stresses in the fluid are

(4) $$\begin{array}{c} \sigma_{f}=-\phi_{f}\;pI, \end{array}$$

where *p* is the pore hydrostatic pressure. In the presence of large regions filled with fluid, the overall fluid shear stresses should be considered too. The stresses in the solid arise from the strain within the solid and from the interaction with the fluid. Under small deformations, and for an isotropic solid, we have

(5) $$\begin{array}{c} \sigma_{s}={\hat{\sigma }}_{s}-\phi_{s}\;pI,\;{\hat{\sigma }}_{s}=\lambda_{s}\mathrm{Tr}\left(\varepsilon \left(u_{s}\right)\right)\;I+2\mu_{s}\;\varepsilon \left(u_{s}\right),\;\varepsilon_{ij}\left(u\right)=\frac{1}{2}\left(\frac{\partial u_{i}}{\partial x_{j}}+\frac{\partial u_{j}}{\partial x_{i}}\right), \end{array}$$

where $u_{s}$ is the displacement vector of the solid biomass; ε(u) the deformation tensor; and $\lambda_{s},\mu_{s},$ the Lamé constants, related to the Young *E* and Poisson ν moduli by $\lambda =\frac{E\nu }{\left(1+\nu )(1-2\nu \right)},$$\mu =\frac{E}{2(1+\nu )}.$

#### 2.2.2. Interaction and Inertial Forces

In most biological samples, the velocities $v_{f}$ and $v_{s}$ are small enough for inertial forces to be negligible: $\rho_{s}a_{s}\approx \rho_{f}a_{f}\approx \rho a\approx 0$, where $a_{s},a_{f},a$ represent the solid, fluid, and composite accelerations. Thus, we will work in a quasi-static deformation regime.

The interaction forces act on the two components. They are opposite in sign and equal in magnitude, as a result, their combined effect vanishes on the tissue. We consider two kinds: filtration resistance and concentration gradients in chemical potentials.

The filtration resistance arises from the interaction between fluid and solid particles. Per unit volume, these forces are $\phi_{s}f_{s}$, $\phi_{f}f_{f}$ and satisfy $\phi_{s}f_{s}+\phi_{f}f_{f}=0.$ In the absence of inertial effects and concentration–viscous couplings $f_{f}=-\alpha q$, where $\alpha (\phi_{f})$ is the resistivity matrix and q the filtration flux. For isotropic elastic solids with isotropic permeability

(6) $$\begin{array}{c} f_{f}=-\frac{1}{k_{h}}q, \end{array}$$

where $k_{h}=\frac{k}{\mu_{f}}$ is the hydraulic permeability, *k* being the permeability of the solid, and $\mu_{f}$ the fluid viscosity. Typically, $k_{h}\left(\phi_{f}\right)=\frac{\phi_{f}^{2}}{\zeta }$, where ζ is a friction parameter often taken to be $\zeta =\frac{\mu_{f}}{\xi {\left(\phi_{s}\right)}^{2}}>0$ and ξ represents the “mesh size” of the matrix network.

The concentration forces in the fluid $\nabla \pi_{f}$ are $\nabla \pi_{f}=-\frac{1}{{\hat{V}}_{f}}\nabla \mu^{f,c},$ where ${\hat{V}}_{f}$ is the molar volume of the fluid and $\mu^{f,c}$ is the concentration contribution to the chemical potential of the fluid $\mu^{f}$. Under isothermal conditions

(7) $$\begin{array}{c} \nabla \mu^{f}={\hat{V}}_{f}\nabla p-\nabla \mu^{f,c}={\hat{V}}_{f}\nabla \left(p-\pi_{f}\right). \end{array}$$

Similar relations hold for concentration forces $\nabla \pi_{s}$ in the solid, which satisfy $\phi_{s}\nabla \pi_{s}+\phi_{f}\nabla \pi_{f}=0.$

### 2.3. Equations of Motion

The theory of mixtures hypothesizes that the motion of each constituent is governed by the usual balance equations, as if it was isolated from the other one. Neglecting inertial terms, and in the absence of external body forces, the momentum balance for the solid and the fluid reads

$$\begin{array}{c} \mathrm{div}\sigma_{s}+\phi_{s}\left(f_{s}+\nabla \pi_{s}\right)=0,\;\mathrm{div}\sigma_{f}+\phi_{f}\left(f_{f}+\nabla \pi_{f}\right)=0. \end{array}$$

Using the expressions for the stress tensors (4) and (5), these equations become

(8) $$\begin{array}{c} \mathrm{div}\;{\hat{\sigma }}_{s}+\phi_{s}\left(-\nabla p+\nabla \pi_{s}\right)+\phi_{s}f_{s}=0,\;\phi_{f}\left(-\nabla p+\nabla \pi_{f}\right)+\phi_{f}f_{f}=0. \end{array}$$

Combining (8), (6) and (3) we obtain the Darcy law in the presence of concentration gradients

(9) $$\begin{array}{c} q=-k_{h}\nabla \left(p-\pi_{f}\right)=\phi_{f}\left(v_{f}-v_{s}\right). \end{array}$$

Adding up Equation (8), we find an equation relating solid displacements and pressure

(10) $$\begin{array}{c} \mathrm{div}\;{\hat{\sigma }}_{s}\left(u_{s}\right)-\nabla p=0. \end{array}$$

The solid velocity is then $v_{s}=\frac{\partial u_{s}}{\partial t}.$ These equations are complemented by the conservation of mass (1) and (2), which now reads as

(11) $$\begin{array}{c} \mathrm{div}\left(v_{s}\right)=-\mathrm{div}\left(q\right)=\mathrm{div}\left(k_{h}\nabla \left(p-\pi_{f}\right)\right). \end{array}$$

The flux (9) can be rewritten as $q=k_{h}\mathrm{div}\left(-{\hat{\sigma }}_{s}+\pi_{f}I\right),$ where $-{\hat{\sigma }}_{s}+\pi_{f}I$ is the swelling stress. In biphasic swelling theory [28], it is customary to work with $p-\pi_{f}=p_{f}$, where $p_{f}$ is associated to the fluid chemical potential by (7) and $\pi_{f}$ is identified with the osmotic pressure created by the concentration of a specific chemical [28,29]. The osmotic pressure in the biofilm is caused by the concentration of EPS produced by the cells and can be taken to be proportional to the volume fraction of solid biomass $\pi_{f}=\Pi \phi_{s},$Π being the osmotic compressibility [11]. Equation (10) then motivates the introduction of effective constitutive laws for the whole mixture of the form [28] $\sigma \left(u\right)={\hat{\sigma }}_{s}\left(u\right)-pI={\hat{\sigma }}_{s}\left(u\right)-\left(p_{f}+\pi_{f}\right)I,$ as usual in poroelastic theory.

### 2.4. Final Equations

Summarizing the main governing equations, we get

(12) $$\begin{array}{cc} \frac{\partial \phi_{s}}{\partial t}+\mathrm{div}\left(\phi_{s}v_{s}\right)=g\left(c_{n}\right)\phi_{s}, & \end{array}$$

(13) $$\begin{array}{cc} \mathrm{div}\left(v_{s}\right)=\mathrm{div}\left(k_{h}\left(\phi_{f}\right)\nabla p_{f}\right), & \end{array}$$

(14) $$\begin{array}{cc} \mu_{s}\Delta u_{s}+\left(\mu_{s}+\lambda_{s}\right)\nabla \left(\mathrm{div}\left(u_{s}\right)\right)=\nabla \left(p_{f}+\pi_{f}\right), & \end{array}$$

(15) $$\begin{array}{c} v_{s}=\frac{\partial u_{s}}{\partial t},\;\pi_{f}=\pi \left(\phi_{s}\right), \end{array}$$

in the region occupied by the biofilm $\Omega_{b}\left(t\right)$, which varies with time. In equilibrium, $q=v_{s}=f_{f}=0.$ At the biofilm boundary, the jumps in the total stress vector and the chemical potential vanish:

$$\begin{array}{c} \left({\hat{\sigma }}_{s}-pI\right)n=\left({\hat{\sigma }}_{s}-\left(p_{f}+\pi_{f}\right)I\right)n=t_{ext},\;p_{f}=p-\pi_{f}=p_{ext}-\pi_{f,ext}, \end{array}$$

when applicable. In this quasi-static framework, the displacements $u_{s}$ depend on time through the motion of the biofilm boundary.

If we need to track the variations of the nutrient concentration, we may use effective continuity equations for chemical concentration in tissues [30,31]. For the limiting concentration $c_{n}$:

(16) $$\begin{array}{c} \frac{\partial c_{n}}{\partial t}+\mathrm{div}\left(v_{f}c_{n}\right)-\mathrm{div}\left(d_{n}\nabla c_{n}\right)=-r_{n}\left(\phi_{s},c_{n}\right),\;r_{n}\left(\phi_{s},c_{n}\right)\;=\;\phi_{s}k_{n}\frac{c_{n}}{c_{n}+K_{n}}, \end{array}$$

where $d_{n}$ is an effective diffusivity [32]. Setting $d_{n,s}$ and $d_{n,f}$, the diffusivities in the biomass and liquid $d_{n}=d_{n,f}\frac{3d-2\phi_{f}\left(d-1\right)}{3+\phi_{f}\left(d-1\right)}$, $d=K_{eq}\frac{d_{n,s}}{d_{n,f}}.$ The source $r_{n}\left(\phi_{s},c_{n}\right)$ represents consumption by the biofilm, $k_{n}$ being the uptake rate and $K_{n}$ the half-saturation constant. Zero-flux boundary conditions are imposed at the air–biofilm interface. Instead, at the agar–biofilm interface, we may impose a constant concentration through a Dirichlet boundary condition. Being more realistic, we couple this diffusion equation to another one defined in the agar substratum $\Omega_{a}\left(t\right)$ with zero source and transmission conditions at the interface [13].

**Figure 3:**
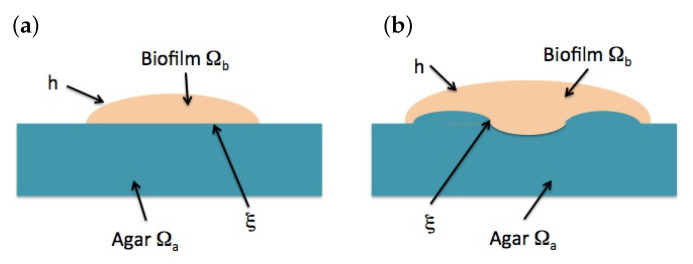
Schematic representation of a biofilm slice, with moving air–biofilm interface *h* and agar–biofilm interface ξ. (**a**) Initial stages: ξ is flat. (**b**) Later evolution: ξ deviates out of a plane.

Solving Equations (12)–(15), studying the evolution of a biofilm also requires tracking the dynamics of the biofilm boundary, see Figure 3. In principle, there are two boundaries of a different nature: the air–biofilm interface and the agar–biofilm interface.

### 2.5. Motion of the Air–Biofilm Interface

During the first stages of biofilm spread, the agar–biofilm interface remains flat, whereas the biofilm reaches a height $x_{3}=h\left(x_{1},x_{2},t\right)$, see Figure 3a. Integrating (2) in the $x_{3}$ direction we obtain $\begin{array}{c} \int_{0}^{h}\frac{\partial (v\cdot {\hat{x}}_{1})}{\partial x_{1}}\;dx_{3}+\int_{0}^{h}\frac{\partial (v\cdot {\hat{x}}_{2})}{\partial x_{2}}\;dx_{3}+\int_{0}^{h}\frac{\partial (v\cdot {\hat{x}}_{3})}{\partial x_{3}}\;dx_{3}=0, \end{array}$ ${\hat{x}}_{1}$, ${\hat{x}}_{2}$ and ${\hat{x}}_{3}$ being the unit vectors in the Cartesian coordinate directions. By Leibniz’s rule

$$\begin{array}{c} \int_{0}^{h}\frac{\partial (v\cdot {\hat{x}}_{i})}{\partial x_{i}}\;dx_{3}=\frac{\partial }{\partial x_{i}}\left[\int_{0}^{h}\left(v\cdot {\hat{x}}_{i}\right)\;dx_{3}\right]-v\cdot {\hat{x}}_{i}{|}_{h}\frac{\partial h}{\partial x_{i}},\;i=1,2. \end{array}$$

Therefore,

(17) $$\begin{array}{c} \begin{array}{c} \frac{\partial }{\partial x_{1}}\left[\int_{0}^{h}\left(v\cdot {\hat{x}}_{1}\right)\;dx_{3}\right]+\frac{\partial }{\partial x_{2}}\left[\int_{0}^{h}\left(v\cdot {\hat{x}}_{2}\right)\;dx_{3}\right]-v\cdot {\hat{x}}_{1}{|}_{h}\frac{\partial h}{\partial x_{1}}-v\cdot {\hat{x}}_{2}{|}_{h}\frac{\partial h}{\partial x_{2}}+v\cdot {\hat{x}}_{3}{|}_{h}=v\cdot {\hat{x}}_{3}{|}_{0}. \end{array} \end{array}$$

Differentiating $x_{3}\left(t\right)=h\left(x_{1}\left(t\right),x_{2}\left(t\right),t\right)$ with respect to time and using $v\cdot {\hat{x}}_{i}=\frac{dx_{i}}{dt}$, i=1,2,3, we find $v\cdot x_{3}{|}_{h}=\frac{dx_{3}}{dt}=\frac{d}{dt}h\left(x_{1}\left(t\right),x_{2}\left(t\right),t\right)=\frac{\partial h}{\partial t}+\frac{\partial h}{\partial x_{1}}\frac{dx_{1}}{dt}+\frac{\partial h}{\partial x_{2}}\frac{dx_{2}}{dt}=\frac{\partial h}{\partial t}+v\cdot x_{1}{|}_{h}\frac{\partial h}{\partial x_{1}}+v\cdot x_{2}{|}_{h}\frac{\partial h}{\partial x_{2}}.$ Inserting this identity in (17), we find the equation

(18) $$\begin{array}{c} \begin{array}{c} \frac{\partial h}{\partial t}+\frac{\partial }{\partial x_{1}}\left[\int_{0}^{h}\left(v\cdot {\hat{x}}_{1}\right)\;dx_{3}\right]+\frac{\partial }{\partial x_{2}}\left[\int_{0}^{h}\left(v\cdot {\hat{x}}_{2}\right)\;dx_{3}\right]=v\cdot {\hat{x}}_{3}{|}_{0}, \end{array} \end{array}$$

where $v\cdot {\hat{x}}_{i}=\frac{du_{s,i}}{dt}-k_{h}\left(\phi_{f}\right)\frac{\partial p_{f}}{\partial x_{i}},i=1,2,3.$ To obtain a closed equation for the height *h* we need to calculate the velocity of the solid $v_{s}=\frac{du_{s}}{dt}$, the modified pressure $p_{f}$ and the volume fraction of fluid from (12) and (13). This equation is able to describe accelerated spread due to osmosis, at least in simplified geometries, as we illustrate next.

From Equation (18), we derive an approximated equation for the early evolution of the height of a circular biofilm, see Appendix A for details and assumptions

(19) $$\begin{array}{c} h_{t}\;-\;\frac{KR}{R_{0}}\frac{e^{3t}}{r}{\left(rh_{rt}h^{3}\right)}_{r}\;-\;\frac{3KR}{2R_{0}}\frac{e^{3t}}{r}{\left(rh_{r}h^{2}h_{t}\right)}_{r}\;-\;\frac{KR}{R_{0}}\frac{e^{3t}}{r}{\left(rh_{r}h^{3}\right)}_{r}\;-\;\frac{3KR}{2R_{0}}\frac{e^{3t}}{r}{\left(rh_{r}h^{3}\right)}_{r}\;-\;\frac{KR_{t}}{R_{0}}\frac{e^{3t}}{r}{\left(rh_{r}h^{3}\right)}_{r}\;=\;0. \end{array}$$

A simplified version

(20) $$\begin{array}{c} h_{t}-K\left(1+\frac{3}{2}\right)Re^{3t}\frac{1}{r}{\left(rh_{r}h^{3}\right)}_{r}=0,\;K=\frac{g\mu_{f}}{3\xi_{\infty }^{2}\mu_{s}{\left(1-\phi_{\infty }\right)}^{2}R_{0}}h_{0}^{3}, \end{array}$$

has self-similar solutions. Restoring dimensions, they take the form

(21) $$\begin{array}{c} h=h_{0}e^{gt}{\left(R/R_{0}\right)}^{-2}f\left(\frac{r}{R}\right)=e^{gt}{\left(R/R_{0}\right)}^{-2}{\left(1-\frac{3}{2}\;\frac{r^{2}}{R^{2}}\right)}^{\frac{1}{3}},\;R=R_{0}{\left(\frac{7}{3}K\left(1+\frac{3}{2}\right)\left(e^{3gt}-1\right)+1\right)}^{\frac{1}{7}}. \end{array}$$

Replacing (1+3/2) by 1 in (21), we recover the self-similar solution found in [11], with $g\mu_{f}$ instead of $\mu_{f}$ ($\mu_{f}$ being the fluid viscosity) and the Lamé coefficient of the solid biomass $\mu_{s}$ instead of the viscosity of the fluid biomass $\mu_{s}$.

**Figure 4:**
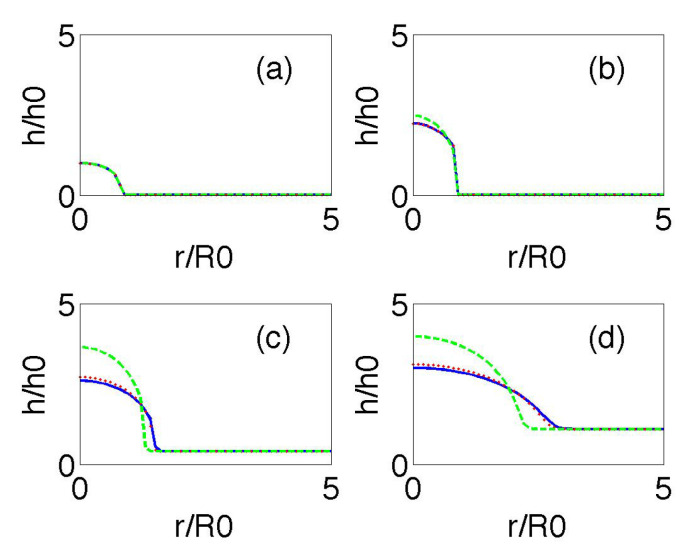
Biofilm height at dimensionless times 0 (**a**), 1 (**b**), 6 (**c**), and 7 (**d**) for $K=10^{-5}$ and $h_{\inf}=10^{-3}.$ The dotted red line and the solid blue line depict the numerical solutions of (20) and (19), respectively, with *R* given by (21) and keeping the same data. We can observe the transition from an initial stage in which increase in biofilm height dominates to a stage with faster horizontal spread. The green line is a reference self-similar approximation.

Figure 4 compares the time evolution of the biofilm height profiles starting from a smoothed version of (21). Notice that (21) only makes sense when $R^{2}>3/2\;r^{2}$, and that the slope diverges at $r=\sqrt{2/3}R$. Experiments show that a thin biofilm layer precedes the advance of the biofilm bulk [11]. We set $h=h_{\infty }>0$ beyond that point. The dashed green line in Figure 4 represents the numerical solution of (20), with *R* given by (21) for $K=10^{-5}$, and $h_{\inf}=10^{-3},$ replacing (1+3/2) by 1 as in [11]. The dotted red line and the solid blue line depict the numerical solution of (20) and (19), respectively, with *R* given by (21) and keeping the same data. They all show the transition from vertical growth to horizontal spreading as time goes on. The effect of the additional time derivatives in (19) is to flatten the profiles.

When the interface biofilm/agar is not flat, but admits a parametrization of the form $x_{3}=\xi \left(x_{1},x_{2},t\right)$, as in Figure 3b, $\begin{array}{c} \int_{\xi }^{h}\frac{\partial (v\cdot {\hat{x}}_{i})}{\partial x_{i}}\;dx_{3}=\frac{\partial }{\partial x_{i}}\left[\int_{\xi }^{h}\left(v\cdot {\hat{x}}_{i}\right)\;dx_{3}\right]-v\cdot {\hat{x}}_{i}{|}_{h}\frac{\partial h}{\partial x_{i}}+v\cdot {\hat{x}}_{i}{|}_{\xi }\frac{\partial \xi }{\partial x_{i}},\;i=1,2. \end{array}$ Repeating the previous computations in the interval [ξ,h], the equation for the biofilm height becomes

(22) $$\begin{array}{c} \begin{array}{c} \frac{\partial h}{\partial t}+\frac{\partial }{\partial x_{1}}\left[\int_{\xi }^{h}\left(v\cdot {\hat{x}}_{1}\right)\;dx_{3}\right]+\frac{\partial }{\partial x_{2}}\left[\int_{\xi }^{h}\left(v\cdot {\hat{x}}_{2}\right)\;dx_{3}\right]=v\cdot {\hat{x}}_{3}{|}_{\xi }-v\cdot {\hat{x}}_{1}{|}_{\xi }\frac{\partial \xi }{\partial x_{1}}-v\cdot {\hat{x}}_{2}{|}_{\xi }\frac{\partial \xi }{\partial x_{2}}. \end{array} \end{array}$$

Knowing ξ, this equation can be solved numerically coupled to (12) and (13).

### 2.6. Motion of the Agar/Biofilm Interface

Equations for the dynamics of the agar–biofilm interface follow using a Von Karman-type approximation, as the thickness of the biofilms is small compared to its radius. Although initially flat, the displacements in the direction orthogonal to the interface may become large. Thus, the linear definition of the strain and stress tensors in (5) is replaced by [33]

(23) $$\begin{array}{ccc} \varepsilon_{i,j} & = & \frac{1}{2}\left(\frac{\partial u_{i}}{\partial x_{j}}+\frac{\partial u_{j}}{\partial x_{i}}+\frac{\partial \xi }{\partial x_{i}}\frac{\partial \xi }{\partial x_{j}}\right)+\varepsilon_{i,j}^{0},\;i=1,2, \end{array}$$

which includes nonlinear terms, as well as residual strains $\varepsilon_{i,j}^{0}$. We denote the in-plane displacements by $u=(u_{1}\left(x_{1},x_{2},t\right),u_{2}\left(x_{1},x_{2},t\right))$ and the out-of-plane displacements of the interface by $\xi (x_{1},x_{2},t)$. The coordinates $\left(x_{1},x_{2}\right)$ vary along the 2D projection of the 3D biofilm structure on the biofilm/agar interface. Equation (14) becomes $\mathrm{div}\hat{\sigma }=\nabla \left(p_{f}+\pi_{f}\right)$, with $\hat{\sigma }$ given by (5) and (23). Formally, this allows us to identify the biofilm with an elastic film growing on a viscoelastic agar substratum. The pressure terms become residual stresses. Then, the interface motion is governed by the equations [13,34]:

(24) $$\begin{array}{c} \frac{\partial \xi }{\partial t}=\frac{1-2\nu_{a}}{2(1-\nu_{a})}\frac{h_{a}}{\eta_{a}}\left[D\left(-\Delta^{2}\xi +\Delta C_{M}\right)+h\frac{\partial }{\partial x_{j}}\left(\sigma_{i,j}\left(u\right)\frac{\partial \xi }{\partial x_{i}}\right)\right]-\frac{\mu_{v}}{\eta_{a}}\xi , \end{array}$$

(25) $$\begin{array}{c} \frac{\partial u}{\partial t}=\frac{h_{a}h}{\eta_{a}}\mathrm{div}\left(\sigma (u)\right)-\frac{\mu_{v}}{\eta_{a}}u, \end{array}$$

where $h_{a}$ is the thickness of the viscoelastic agar substratum and $\mu_{v}$, $\nu_{a}$, and $\eta_{a}$ its rubbery modulus, Poisson ratio, and viscosity, respectively. The tensor σ is given by

(26) $$\begin{array}{c} \begin{array}{c} \sigma_{11}=\frac{E}{1-\nu^{2}}\left(\varepsilon_{11}+\nu \varepsilon_{22}\right)+\sigma_{11}^{0},\;\sigma_{12}=\frac{E}{1+\nu }\varepsilon_{12}+\sigma_{12}^{0},\;\sigma_{22}=\frac{E}{1-\nu^{2}}\left(\varepsilon_{22}+\nu \varepsilon_{11}\right)+\sigma_{22}^{0}, \end{array} \end{array}$$

with ε defined in (23); ν and *E* being the Poisson and Young moduli of the biofilm (5), respectively; and $\sigma^{0}$ represents the residual stresses. The bending stiffness is $D=\frac{Eh^{3}}{12(1-\nu^{2})}$, *h* being the initial biofilm thickness. Here, the first Equation (24) describes out-of-plane bending ξ, and the second one (25) governs in-plane stretching for the displacements $u=(u_{1},u_{2})$. Modified equations taking into account possible spatial variations in the elastic moduli are given in [35].

To identify the relevant scales governing the evolution of the agar–biofilm interface we nondimensionalize (24) and (25). Making the change of variables $\hat{x}=\frac{x}{R},$$\hat{u}=\frac{u}{R},$$\hat{\xi }=\frac{\xi }{h},$$\hat{\sigma }=\frac{\sigma }{E},$$\hat{t}=\frac{t}{T},$ where *R* is the approximate biofilm radius, and setting R=γh, the dimensionless equations become

(27) $$\begin{array}{c} \frac{\partial \hat{\xi }}{\partial \hat{t}}=\left[12\left(1-\nu^{2}\right)\gamma^{2}\frac{\partial }{\partial {\hat{x}}_{j}}\left({\hat{\sigma }}_{i,j}\left(\hat{u}\right)\frac{\partial \hat{\xi }}{\partial {\hat{x}}_{i}}\right)+\left(-\Delta_{\hat{x}}^{2}\hat{\xi }+\Delta_{\hat{x}}{\hat{C}}_{M}\right)\right]-T\frac{\mu_{a}}{\eta_{a}}\hat{\xi }, \end{array}$$

(28) $$\begin{array}{c} \frac{\partial \hat{u}}{\partial \hat{t}}=\tau \;{\mathrm{div}}_{\hat{x}}\hat{\sigma }\left(\hat{u}\right)-T\frac{\mu_{a}}{\eta_{a}}\hat{u}, \end{array}$$

where $T=\frac{2(1-\nu_{v})}{1-2\nu_{v}}\frac{\eta_{v}h}{h_{v}}\frac{12\left(1-\nu^{2}\right)\gamma^{4}}{E}=\tau \frac{\eta_{v}h}{h_{v}E}\gamma^{2},$$\tau =24\frac{\left(1-\nu_{v}\right)}{\left(1-2\nu_{v}\right)}\left(1-\nu^{2}\right)\gamma^{2}.$ Wrinkled structures develop when the nonlinear terms are large enough, therefore $\gamma =\frac{R}{h}$ must be large enough.

**Figure 5:**
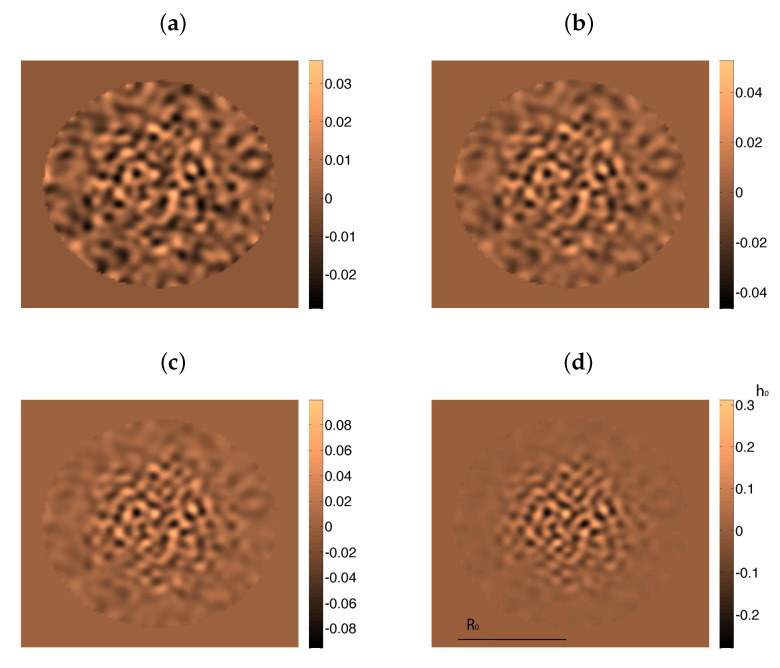
Wrinkle formation and coarsening in a growing film with residual stresses computed from analytical formulas for the pressures. As the approximation breaks down, the height of the central wrinkles increases much faster than the height of the outer ones, which blur in comparison. Snapshots taken at times (**a**) 1.8/g, (**b**) 2/g, (**c**) 2.2/g, and (**d**) 2.4/g, starting from a randomly perturbed biofilm of radius $R_{0}=10^{-3}$ m and height $h_{0}=10^{-4}$ m. The radius does not vary significantly during this time, whereas the height becomes of the order of the radius at the end. Parameter values: 1/g=2.3 hours, $\mu_{f}=8.9\times 10^{-4}$ Pa·s at 25^o^, $\xi_{\infty }=70$ nm, $\phi_{\infty }=0.2$, $h_{a}=100h_{0}$, Π=30 Pa (taken from [11]), E=25 kPa (taken from [12]), ν=0.4, $\mu_{s}=8.92$ kPa, $\nu_{a}=0.45$, $\eta_{a}=1$ kPa·s, $\mu_{a}=0$, $h_{inf}=h_{0}/10$.

The residual stresses $\sigma^{0}$ in (26) can be estimated averaging the osmotic and fluid pressure contributions to the three-dimensional biofilm. If the solution of (12)–(15) in the biofilm $\Omega_{b}\left(t\right)$ is known, $\sigma_{ij}^{0}$ could be estimated from

(29) $$\begin{array}{c} -\int_{x_{3}=\xi }^{x_{3}=h}\left[\left(p_{f}+\pi_{f}\right)I\right]\;dx_{3},\;i,j=1,2, \end{array}$$

where $x_{3}=h$ and $x_{3}=\xi$ define the two biofilm interfaces with air and agar, see Figure 3. Analytical approximations (19)–(21) of the the biofilm height *h* in early stages of the biofilm evolution allow for simple simulations of the onset of wrinkle formation. Figure 5 uses these asymptotic profiles to compute pressures and velocities by means of (A11)–(A14). Starting from an initially flat biofilm, (29) suggests that we should consider stress profiles of the form $-Ah^{2}/2-ARh-\Pi \phi_{\infty }$, where $A=\frac{g\mu_{f}}{\xi_{\infty }^{2}{\left(1-\phi_{\infty }\right)}^{2}},$ which are nondimensionalized dividing by *E*. The first two terms reflect the stresses due to growing height and radius, whereas the last one accounts for the osmotic pressure. Inserting these residual stresses in (24)–(26) we generate small inhomogeneities and wrinkles in Figure 5. However, these approximations neglect spatial variations in concentrations, as well as changes in cell behavior, and therefore, in stresses and pressures. Therefore, the patterns display soon an unrealistic behavior, with wrinkles excessively growing in the central region.

Solving the full set of coupled equations we have derived is very costly and faces severe numerical difficulties at contact points. Alternatively, we may set $\sigma^{0}=0$ and work with the residual strains $\varepsilon^{0}$ in (23), which can be related to growth tensors created by stochastic cell processes as we discuss next.

## 3. Incorporating Cellular Behavior

Cells within a biofilm differentiate to perform different tasks, and can deactivate due to lack of resources or die as a result of biochemical stress and waste accumulation [10,12]. Such variations in the biofilm microstructure affect the overall shape [13]. Cell activity enters the previous deterministic model through the biomass creation term $g\left(c_{n}\right)\phi_{s}$ in (12), the nutrient consumption term $r_{n}\left(\phi_{s},c_{n}\right)$ in (16), and the residual stresses $\sigma^{0}$ in (26). However, this does not account for cell death, cell deactivation and cell differentiation.

**Figure 6:**
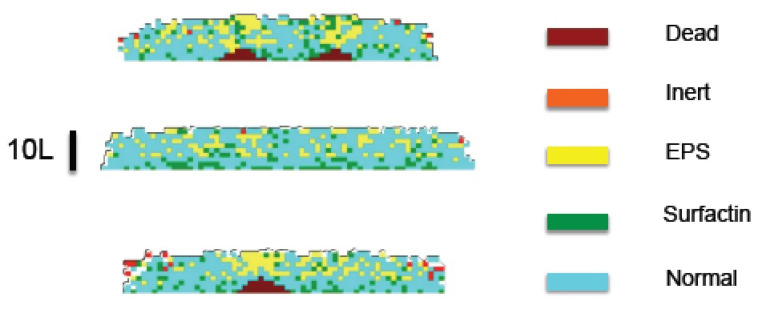
Layered distribution of dead, active, and inert cells, as illustrated by slices of a growing biofilm. Dead cells appear at the bottom of three peaks present in the initial biofilm seed.

Differentiation implies changes in phenotype while preserving the same genotype. For *B. subtilis* biofilms, the differentiation chain through which different cell types originate is established in [10], see Figure 6. Initially, we have a population of similar alive cells glued together in a matrix, most of which have lost their individual motility. All of them secrete ComX. If the concentration of ComX becomes large enough, some cells differentiate and start producing surfactin, losing their ability to reproduce. For large enough surfactin concentrations, other normal cells differentiate and become EPS producers. These cells reproduce more slowly than normal cells. Cells may also die due to biochemical stresses [12], preferentially at high-density regions, such as the agar–biofilm interface. In the upper regions of the biofilm, depletion of resources may trigger deactivation of cells, which become spores. Undifferentiated cells retaining some motility are restricted to the bottom edges [10].

To a large extent, these processes have a random character. Hybrid models combine stochastic descriptions of cellular processes with continuous equations for other relevant fields. This allows us to consider the inherent randomness of individual bacterial behaviors as well as local variations [10,36]. We will explain how to introduce cell variability in the mixture model next.

### 3.1. Cellular Automata and Dynamic Energy Budget

In a cellular automata approach, space is divided in a grid of cubic tiles. This grid is used to discretize all the continuous equations: concentrations, deformations, pressures, etc. To simplify geometrical considerations, we initially assume that each tile can contain one bacterium at most. We describe bacterial metabolism using a dynamic energy budget framework [9,37]. According to this theory [37], cells create energy from nutrients/oxygen, which they use for growth, maintenance, and product synthesis. Damage-inducing compounds can cause death. The metabolism of each cell $C_{j}$ is described by the system:

(30) $$\begin{array}{c} \begin{array}{cc} \frac{de_{j}}{dt}=\nu^{\prime }\left(f-e_{j}\right), & f=\frac{c_{n}}{c_{n}+K_{n}},\;\nu^{\prime }=\nu e^{-\gamma \varepsilon },\\ \frac{dv_{j}}{dt}=\left(r_{j}\frac{a_{j}}{a_{M}}-h_{j}\right)v_{j}, & r_{j}={\left(\frac{\nu^{\prime }e_{j}-mg}{e_{j}+g}\right)}^{+},\\ \frac{dv_{e,j}}{dt}=\left(1-\alpha \right)r_{e,j}v_{j}, & r_{e,j}=kr_{j}+k^{\prime },\\ \frac{dq_{j}}{dt}=e_{j}\left(s_{G}\rho v_{j}\;q_{j}\;+\;h_{a}\right)\left(\nu^{\prime }\;-\;r_{j}\right)-\left(r_{j}\;+\;r_{e,j}\right)q_{j},\\ \frac{dh_{j}}{dt}=q_{j}-\left(r_{j}+r_{e,j}\right)h_{j},\\ \frac{dp_{j}}{dt}=-h_{j}p_{j}, & p_{j}\left(0\right)=1,\\ \frac{da_{j}}{dt}=\left(r_{j}+r_{e,j}\right)\left(1-\frac{a_{j}}{a_{M}}\right), \end{array} \end{array}$$

where the cell variables are their energy $e_{j}\left(t\right)$, volume $v_{j}\left(t\right)$, produced volumes of EPS $v_{e,j}\left(t\right)$, acclimation $a_{j}\left(t\right)$, damage $q_{j}\left(t\right)$, hazard $h_{j}\left(t\right)$, and survival probability $p_{j}\left(t\right)$, for j=1,…,N, *N* being the total number of cells. These equations are informed by the value of continuous fields at the cell location x (the tile of the cellular automata grid containing the cell): nutrient concentration $c_{n}\left(x,t\right)$, polymeric matrix concentration $c_{e}\left(x,t\right)$, surfactin concentration $c_{s}\left(x,t\right)$, ComX concentration $c_{cx}\left(x,t\right)$, and environmental degradation ε(x,t), which are governed by

(31) $$\begin{array}{c} \begin{array}{c} \frac{dc_{n}}{dt}=-\nu^{\prime }f\rho \sum_{j}v_{j}\delta_{j}+\mathrm{div}\left(d_{n}\nabla c_{n}\right)-\mathrm{div}\left(v_{f}c_{n}\right),\\ \frac{dc_{cx}}{dt}=\rho \sum_{j}r_{cx,j}v_{j}\delta_{j}+\mathrm{div}\left(d_{cx}\nabla c_{cx}\right)-\mathrm{div}\left(v_{f}c_{cx}\right),\\ \frac{dc_{s}}{dt}=\rho \sum_{j}r_{s,j}v_{j}\delta_{j}+\mathrm{div}\left(d_{s}\nabla c_{s}\right)-\mathrm{div}\left(v_{f}c_{s}\right),\\ \frac{dc_{e}}{dt}=\alpha \rho \sum_{j}r_{e,j}v_{j}\delta_{j}+\mathrm{div}\left(d_{e}\nabla c_{e}\right)-\mathrm{div}\left(v_{f}c_{e}\right),\\ \frac{d\varepsilon }{dt}=\nu_{\varepsilon }\sum_{j}\left(r_{j}+\nu_{m}m\right)v_{j}\delta_{j}+\mathrm{div}\left(d_{\varepsilon }\nabla \varepsilon \right)-\mathrm{div}\left(v_{f}\varepsilon \right). \end{array} \end{array}$$

The parameters ν, *m*, *g*, $a_{M}$, and ρ are the energy conductance, the maintenance rate, the investment ratio, the target acclimation energy, and mass density for the bacteria under consideration, respectively. Other coefficients are the multiplicative stress coefficient $s_{G}$, the maintenance respiratory coefficient $\nu_{m}$, the noncompetitive inhibition coefficient $K_{v}$, and the environmental degradation coefficients γ and $\nu_{\varepsilon }$. The parameters $d_{n}$, $d_{cx}$, $d_{s}$, $d_{e}$, and $d_{\varepsilon }$ stand for diffusivities. The Dirac masses $\delta_{j}$ are equal to 1 at the location of cell $C_{j}$ and zero outside.

The production rates $r_{s,j}$ and $r_{e,j}$ are zero, except when the cell is a surfactin producer, or an EPS producer, respectively. In the latter case, $r_{e,j}=kr_{j}+k^{\prime }$, where *k* and $k^{\prime }$ correspond to constants controlling the chemical balances for polymer production. The parameter α∈[0,1] regulates the fraction of produced polymer that remains in a monomeric state and diffuses as $c_{e}$, instead of becoming part of larger chains that remain attached to the cells forming the matrix $v_{e}$.

In this framework, bacteria $C_{j}$ die with probability $1-p_{j}$. Taking a cellular automata view, we modify the cell nature according to selected probabilities, which are defined in terms of concentration values at the cell location. A normal bacterium becomes a surfactin producer with probability $p_{cx}=\frac{c_{cx}}{c_{cx}+K_{cx}^{*}}$ and an EPS producer with probability $p_{e}=\frac{c_{s}}{K_{s}^{*}+c_{s}}\left(1-\frac{c_{n}}{K_{n}^{*}+c_{n}}\right)$. Cells become become inert with probability $p_{i}=1-\frac{c_{n}}{K_{n}^{*}+c_{n}}$. A non-surfactin-producer whose volume has surpassed a critical volume for division, divides with probability $p_{d}=\frac{c_{n}}{K_{n}^{*}+c_{n}}$. Figure 6 represents the cell type distribution for a growing biofilm. The simulation started from a circular seed with a diameter of 60 cells, and nonuniform height. Each colored box in the slices represents one cell. The brown areas representing dead cells appear at the bottom of three initial peaks. We set $\frac{k_{n}L^{2}}{d_{n}K_{n}}=0.01$, $\frac{k_{cx}L^{2}}{d_{cx}K_{cx}}=0.01,$ and $\frac{k_{s}L^{2}}{d_{s}K_{s}}=0.8.$, where *L* is a reference length representing the tile size (approximately the bacterium size 2 μm).

**Figure 7:**
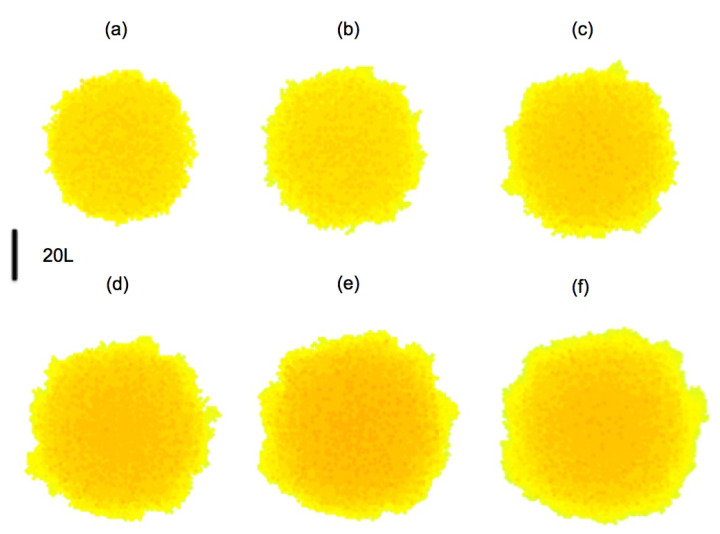
Top view of the evolution of a biofilm seed formed by two layers of cells with a diameter of 40 cells, depicted at steps: (**a**) 45; (**b**) 90; (**c**) 135; (**d**) 180; (**e**) 225; (**f**) 270. Darkest colors correspond to layers of increasing height up to 10 cells. Contour undulations develop as the biofilm spreads.

In principle, when a bacterium divides, the daughters occupy the space left by it, while pushing the other bacteria. Dealing with the geometrical aspects of arrangements of dividing bacteria is a complicate issue for which different approaches have been explored [4,5]; it is out of the scope of the present work. For simplicity, we consider here that space is partitioned in a grid of cubic tiles, as explained earlier, and this grid is used to discretize all the continuous equations for concentrations, displacements, pressures, etc. Each tile may contain at maximum one bacterium, the tile size is the maximum size a bacterium may attain. Once a bacterium divides, one daughter remains in the tile, whereas the other occupies a random neighboring tile, either empty, or containing a dead cell, which is reabsorbed. In the absence of them, it will push neighboring cells in the direction of minimal mechanical resistance, that is, minimal distance to air. The resulting collection of tiles defines the new $\Omega_{b}$. Figure 7 represents the evolution of a growing biofilm seed considering only division processes with $c_{n}\left(0\right)=K_{n}$ and $\frac{k_{n}L^{2}}{d_{n}K_{n}}=8$. Notice the development of contour undulations.

The resulting full computational model would proceed as follows.

*Initialization*.

- We set an initial distribution of *N* bacteria characterized by their energies $e_{j}\left(0\right)$, volumes $v_{j}\left(0\right)$, damage $q_{j}\left(0\right)$, hazard $h_{j}\left(0\right)$, acclimation $a_{j}\left(0\right)$, and attached EPS volume $v_{e,j}\left(0\right)$, j=1,…,N.
- Each bacterium is initially classified as normal, surfactin producer, EPS producer, or inert. Bacteria are distributed in the tiles x of the grid. The empty space around them is filled with water and dissolved substances. In this way, we may compute the volume fractions of biomass $\phi_{s}\left(x,0\right)$ and fluid $\phi_{f}\left(x,0\right)$ in each tile x, as well as the osmotic pressure Π(x,0). The pressure p(x,0) is obtained from (13) with $v_{s}=0$ and $\sigma^{0}\left(x,0\right)$ from (29).
- We compute stationary solutions of the Equation (31) for $v_{f}=0$ by a relaxation numerical scheme. All except the equation for $c_{n}$ are solved using the grid defining $\Omega_{b}\left(0\right)$ with no flux boundary conditions. The equation for $c_{n}$ is solved in the biofilm–agar domain, that is, $\Omega_{b}\left(0\right)\cup \Omega_{a}\left(0\right)$, imposing continuity of concentrations and fluxes at the agar–biofilm interface and no flux boundary conditions at the air–biofilm interface.

*Evolution with a time step dt: From time t−dt to t*.

- We update ξ(t) using (24)–(29). Keeping the grid tile fixed, we shift the contains of the tiles upwards of downwards to reflect the evolution of ξ(t) when the deflections are large enough.
- We update the cellular fields solving (30) with the stationary concentration values for (31). The time derivatives in (31) are small, so that time evolution is driven by the source terms reflecting cell activity and changes in the biofilm boundaries.
- We update the bacterial status checking whether normal bacteria become surfactin or EPS producers, whether any of them deactivates or dies, and whether they divide, with the probabilities assigned to each situation. In case a bacterium divides, we reallocate the newborn cell.
- In the resulting biofilm configuration $\Omega_{b}\left(t\right)$, we compute the volume fractions of biomass $\phi_{s}\left(x,t\right)$ and fluid $\phi_{f}\left(x,t\right)$ in each tile. This also provides the osmotic pressure $\pi_{f}\left(x,t\right).$ The fluid pressure p(x,t) is obtained from (13), the displacements $u_{s}\left(x,t\right)$ from (13), and $\sigma^{0}\left(x,t\right)$ from (29). The solid velocities are approximated by $v_{s}\left(x,t\right)∼\left(u_{s}\left(x,t\right)-u_{s}\left(x,t-dt\right)\right)/dt$. Then, the fluid velocity is given by (9).
- We yet need to take into account water absorption from agar. To do so, we solve $\frac{\partial \phi_{f}}{\partial t}+\mathrm{div}\left(v_{f}\phi_{f}\right)=0$ in the biofilm/agar system. Alternatively, we can solve only in the biofilm, using $\phi_{f}=0$ at the biofilm/agar interface and $\frac{\partial \phi_{f}}{\partial n}=\frac{h}{R}\phi_{f}$ for boundary conditions, where *h* and *R* are reference values for the biofilm height and radius. Then, we revise the biofilm configuration, creating water tiles with probability $\phi_{f}$ and shifting the contains of the neighbouring tiles. This provides the final biofilm configuration $\Omega_{b}\left(t\right)$, that is, the occupied tiles, their contents, the bacterial status and fields, as well as the values of the continuous fields at each tile.

**Figure 8:**
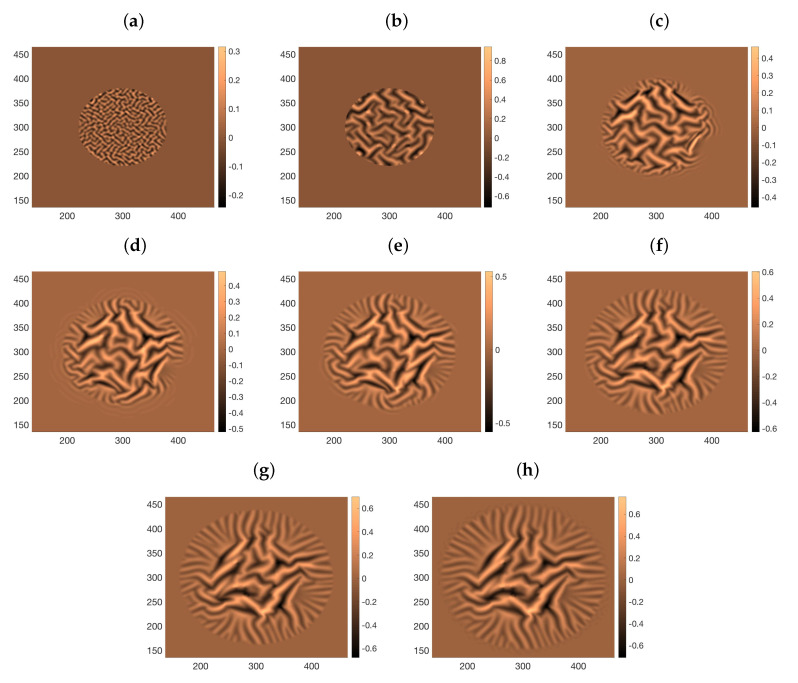
Snapshots of wrinkle formation, coarsening and branching as a circular biofilm expands following (27) and (28) and using an empirical fit to the residual stresses generated by cellular processes. The biofilm has Poisson ratio ν=0.5 and Young modulus E=25 kPa. The Poisson ratio and rubbery modulus of the substratum are $\nu_{v}=0.45$, $\mu_{v}=0,$ and γ=16. (**a**) $26\frac{T}{\tau }$ s; (**b**) $260\frac{T}{\tau }$ s; (**c**) $520\frac{T}{\tau }$ s; (**d**) $780\;\frac{T}{\tau }$ s; (**e**) $1040\frac{T}{\tau }$ s; (**f**) $1300\frac{T}{\tau }$ s; (**g**) $1560\frac{T}{\tau }$ s; (**h**) $1820\frac{T}{\tau }$ s;

This process mixes the stochastic evolution of some cellular processes with continuous equations for a number of fields. In case, any of the fields computed from the tile configuration is not smooth enough to solve the required partial differential equations, we filter it using a total variation based filter introduced in [18] to avoid such artifacts. Figure 8 depicts the formation, coarsening, and branching of wrinkles in an spreading biofilm when the residual stresses are fitted by an empirical circular front approximation of magnitude −0.1 advancing one grid box every 14/τ seconds.

This hybrid model also introduces a number of parameters that should be calibrated to experimental data, not yet available. The parameters appearing in the dynamic energy budget equations have been fitted to experimental measurements for *Pseudomonas aeruginosa* biofilms under the action of antibiotics [9]; fitting to *Bacillus subtilis* would require new specific experiments.

### 3.2. Balance Equation Approach

**Figure 9:**
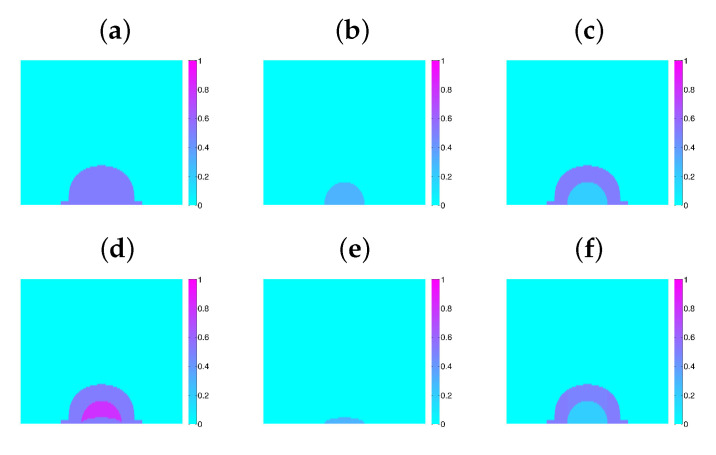
Effect of the presence of dead regions in liquid transport. Initial volume fractions: (**a**) water, (**b**) dead cells, and (**c**) alive cells. Snapshot showing dead cell reabsorption and water accumulation in the originally dead regions at a later time: volume fractions of (**d**) water, (**e**) dead cells, and (**f**) alive cells.

The macroscopic effect of the presence of differentiated bacteria can partially be understood by means of additional balance equations, inserting specific information in them. Let us set $\phi_{a}$ and $\phi_{d}$ as the volume fraction of active and dead cells, respectively. We introduce an additional volume fraction of inert cells $\phi_{i}$, in such a way that $\phi_{s}=\phi_{a}+\phi_{i}+\phi_{d}.$ The balance equations become

(32) $$\begin{array}{c} \frac{\partial \phi_{a}}{\partial t}+\mathrm{div}\left(\phi_{a}v_{s}\right)=\left[g\left(c_{n}\right)-g_{w}\left(c_{w}\right)-g_{i}{\left(c_{n}\right)}^{+}\right]\phi_{a}+g_{i}{\left(c_{n}\right)}^{-}\phi_{i}, \end{array}$$

(33) $$\begin{array}{c} \frac{\partial \phi_{d}}{\partial t}+\mathrm{div}\left(\phi_{d}v_{s}\right)=g_{w}\left(c_{w}\right)\phi_{a}-k_{r}\phi_{d}, \end{array}$$

(34) $$\begin{array}{c} \frac{\partial \phi_{i}}{\partial t}+\mathrm{div}\left(\phi_{i}v_{s}\right)=g_{i}{\left(c_{n}\right)}^{+}\phi_{a}-g_{i}{\left(c_{n}\right)}^{-}\phi_{i}, \end{array}$$

(35) $$\begin{array}{c} \frac{\partial \phi_{f}}{\partial t}+\mathrm{div}\left(\phi_{f}v_{f}\right)=-r_{s}\left(\phi_{a},\phi_{d},c_{n}\right), \end{array}$$

where $r_{s}\left(\phi_{a},\phi_{d},c_{n}\right)=g\left(c_{n}\right)\phi_{a}-k_{r}\phi_{d}$, $k_{r}$ being the rate of reabsorption of dead cells and $c_{w}$ the concentration of waste. The concentration of nutrients still obeys (16), replacing $\phi_{s}$ by $\phi_{a}$ in the consumption term, whereas the concentration of waste $c_{w}$ obeys a similar reaction–diffusion equation with source $r_{w}\left(\phi_{a}\right)=k_{w}\phi_{a}$, $k_{w}>0$. Here, $g_{i}\left(c_{n}\right)$ is positive for small enough values of $c_{n}$ and negative otherwise. For instance, we might take $g_{i}\left(c_{n}\right)=\alpha -\frac{c_{n}}{c_{n}+K_{n}},$α∈(0,1). We assume that dead and alive cells move with the velocity of the solid biomass $v_{s}$. Adding up Equations (32)–(35), we recover the relations (2) and (13). The displacements of the solid biomass $u_{s}$ still obey (14) with two modifications. First, the osmotic pressure $\pi_{f}$ depends only on the fraction of cells producing EPS, which must be alive. Thus, $\pi_{f}=\pi \left(\phi_{a}\right).$ Second, the elastic constants $\lambda_{s}$ and $\mu_{s}$ may vary spatially in case necrotic regions containing a large density of dead cells or swollen regions appear. We focus here on the effect of necrotic regions on liquid transport within the biofilm. Figure 9 illustrates water accumulation in regions with an initially high volume fraction of dead cells.

To investigate the spatial distribution of cells secreting autoinducers, we introduce additional volume fractions of active cells $\phi_{a}=\phi_{u}+\phi_{surf}+\phi_{eps},$ where $\phi_{surf}$ and $\phi_{eps}$ stand for cells producing surfactin and EPS respectively, whereas $\phi_{u}$ are undifferentiated active cells. The balance equations governing the different subpopulations are

(36) $$\begin{array}{ccc} \frac{\partial \phi_{u}}{\partial t}+\mathrm{div}\left(\phi_{u}v_{s}\right) & = & g\left(c_{n}\right)\phi_{u}+g_{e}\left(c_{n}\right)\phi_{eps}-\left[g_{c}\left(c_{cx}\right)+g_{s}\left(c_{surf}\right)\right]\phi_{u} \end{array}$$

(37) $$\begin{array}{ccc} \frac{\partial \phi_{surf}}{\partial t}+\mathrm{div}\left(\phi_{surf}v_{s}\right) & = & g_{c}\left(c_{cx}\right)\phi_{u}, \end{array}$$

(38) $$\begin{array}{ccc} \frac{\partial \phi_{eps}}{\partial t}+\mathrm{div}\left(\phi_{eps}v_{s}\right) & = & g_{s}\left(c_{surf}\right)\phi_{u}, \end{array}$$

where $g_{e}\left(c_{n}\right)=\beta g\left(c_{n}\right)$, β∈(0,1), $g_{s}\left(c_{surf}\right)=k_{surf}^{*}\frac{c_{surf}}{c_{surf}+K_{surf}^{*}}$, and $g_{c}\left(c_{cx}\right)=k_{cx}^{*}\frac{c_{cx}}{c_{cx}+K_{cx}^{*}}$. The autoinducer concentrations are governed by balance equations of the form (16) with sources $r_{surf}=k_{surf}\left(1-\frac{c_{surf}}{c_{surf}+K_{surf}}\right)$ and $r_{cx}=k_{cx}\left(1-\frac{c_{cx}}{c_{cx}+K_{cx}}\right)$, respectively, as well as no-flux boundary conditions at the biofilm boundaries.

If we consider dead and inert cells too, systems (32)–(35) should be updated replacing $g\left(c_{n}\right)\phi_{a}$ by $g\left(c_{n}\right)\phi_{u}+g_{e}\left(c_{n}\right)\phi_{eps}$ in Equation (32) and in the definition of $r_{s}$ for Equation (35). Likewise, systems (36)–(38) should be updated including transfer to and from the inert status.

## 4. Discussion

Growth of cellular aggregates involves mechanical, chemical, and cellular processes acting in different time scales. Bacterial biofilms provide basic environments to test hypotheses and mathematical models against experimental observations. Recent experimental work with *Bacillus subtilis* reveals a host of phenomena during biofilm formation and spread. Different approaches have been exploited to account for different aspects: thin film equations and two-phase flow models for accelerated spread caused by osmosis [11], elasticity theory for the onset of wrinkle formation [12,35], Von Karman-type approximations for wrinkle branching [13,16], and Neo Hookean models for contour undulations and fold formation [17]. In principle, poroelastic models allow to consider liquid transport and elastic deformation in a unified way [18], though detachment and blister formation require further developments [15]. Current models take mainly a deterministic point of view, thus, random cell behavior linked to fluctuations is poorly accounted for. However, cell differentiation [10] to incorporate new phenotypes performing new tasks, such as autoinducer and EPS matrix production, plays a key role in biofilm development. Elementary cellular automata approaches were implemented in [13,18] and used to generate nonuniform residual stresses partially defining the biofilm shape. Here, we develop a hybrid computational model, combining a solid—fluid mixture description of mechanical and chemical processes with a dynamic energy budget based cellular automata approach to cell metabolism.

Cellular automata representations are convenient from a computational point of view, as they allow for simple rules to transfer information between individual cells and the film. However, they provide too crude a representation of bacterial geometry. In our framework, this representation could be improved by resorting to different agent based models. Individual-based models, originally developed to study biofilms in flows [20,21], have recently been adapted to describe biofilms spreading over air–agar interfaces and solid–semisolid interfaces [4,5]. Similarly, immersed boundary methods introduced to study bodies immersed in fluids are being extended to study biofilm spread in flows [38] and at interfaces [39]. We could resort to Individual based or Immersed boundary approaches for a better description of bacterial geometry and their spatial arrangements.

Working with biofilms spreading on an air–agar interface, we have chosen to represent the presence of small fractions of polymeric matrix in an effective way, as done in previous related work [11,22]. The biomass formed by bacteria and polymeric threads is considered one phase [11], with elastic properties as in [22]. The liquid transporting dissolved chemicals is considered a fluid phase. Production of EPS also affects internal liquid flow by osmosis, mechanism we include in our equations for the fluid phase. Depending on the relative fractions and the properties of each phase as well as the characteristic times and lengths, the whole system may display an elastic, fluid, viscoelastic, or truly poroelastic behavior [25,26]. This formulation allows to derive effective equations for the dynamics of the interfaces including the effect of biomass growth, fluid, and osmotic pressures through residual strains and stresses. Resorting to individual-based or immersed boundary representations of cells, we might describe the polymeric matrix as a network of threads instead [4,38], but we should define heuristic rules for their behavior.

Constructing numerical solutions of the full model is a computational challenge, out of the scope of the present work. Instead, we construct numerical solutions, in particular, geometries, guided often by asymptotic simplifications. In this way, we show that the model is able to reproduce behaviors experimentally observed: accelerated spread due to water intake [11,15], wrinkle formation and branching [12,14,15], layered distributions of differentiated cells [10], development of undulations in the contour [15,17], and appearance of regions containing a high volume fraction of water [14,15]. Existing models are devised to explain specific behaviors in relation with particular experiments. An advantage of our approach is that a single model can be used to display all those behaviors and to simulate or even analyze under which conditions they are observed, as the model allows for asymptotic analysis in specific situations. The partial study of different phenomena also suggests empirical expressions for magnitudes representing cellular activities required by the mixture model, such as source terms or residual stresses, which can be inserted in it to reduce computational costs. Our simulations of biofilm spread and wrinkle formation use parameter values experimentally measured for *Bacilus subtilis* biofilms in [11,12], producing reasonable qualitative and quantitative results. However, the parameters for the dynamic energy budget systems for cell metabolism, as well as those appearing in the concentration equations are taken from *Pseudomonas aeruginosa* studies [9]. The probability laws for the cellular automata model and the balance equations for differentiated cell populations involve additional unknown parameters. Thus, our model involves a collection of parameters that should be fitted to experimental data, specially as far as cell metabolism is concerned. Experimental measurements of bacterial dynamics allowing to fit such parameters are yet missing.

## Appendix A

Here, we derive approximate expressions for the volume fractions, velocity, and pressure fields, as well as equations for the height, by considering a simplified version of the equations presented in Section 2 for a slice in the plane $x_{1}x_{3}$, as in Figure 3a.

Only the components $x_{1}$ and $x_{3}$ of the variables are relevant. To simplify the notation, in this section we take $\phi =\phi_{s}$, $p_{f}=p$, $\pi_{f}=\pi =\Pi \phi$, $u_{s}=u$, $v_{s}=v$. The subsequent arguments follow those in [11] for biphasic fluid mixtures, with adequate modifications when necessary.

Following the work in [11], we assume that *g* is approximately constant and $\phi_{t}<<g\phi$. Then, the governing equations become

(A1) $$\begin{array}{c} \begin{array}{c} \mathrm{div}\left(v\phi \right)=g\phi ,\;\mathrm{div}\left(v\right)=\mathrm{div}\left[\frac{{\left(1-\phi \right)}^{2}}{\zeta }\nabla p\right],\;\mu_{s}\Delta u+\left(\mu_{s}+\lambda_{s}\right)\nabla \left(\mathrm{div}\left(u\right)\right)=\nabla \left(p+\Pi \phi \right), \end{array} \end{array}$$

where $v=\frac{\partial u}{\partial t}.$ Recall that $\zeta =\frac{\mu_{f}}{\xi {\left(\phi \right)}^{2}}$, we will set $\zeta =\frac{\mu_{f}}{\xi_{\infty }^{2}}$ with $\xi_{\infty }=\xi \left(\phi_{\infty }\right),$ $\phi_{\infty }$ being a background value. We fix the displacements at the biofilm/agar interface $x_{3}=0$ and impose no stress conditions at the biofilm–air interface $x_{3}=h$, assuming that the normal vector n∼(0,1):

(A2) $$\begin{array}{c} \begin{array}{c} \left(u_{1},u_{3}\right){|}_{x_{3}=0}=\left(0,0\right),\;\sigma_{13}{|}_{x_{3}=h}=\mu_{s}\left(u_{1,x_{3}}+u_{3,x_{1}}\right){|}_{x_{3}=h}=0,\\ \sigma_{33}{|}_{x_{3}=h}=\left[-\left(\pi_{f}+p_{f}\right)+2\mu_{s}u_{3,x_{3}}+\lambda_{s}\mathrm{div}\left(u\right)\right]{|}_{x_{3}=h}=-{\left(\pi_{f}+p_{f}\right)}_{ext}. \end{array} \end{array}$$

Next, we nondimensionalize setting $u_{i}=U_{i}u_{i}^{\prime }$, $v_{i}=V_{i}v_{i}^{\prime }$, $x_{1}=R_{0}x_{1}^{\prime }$, $x_{3}=hx_{3}^{\prime }$, $R=R_{0}R^{\prime }$, $p=Pp^{\prime }$, with $h/R_{0}<<1$. In practice, the height $h=h(x_{1},t)$ depends on $x_{1}$ and *t* and the reference radius $R_{0}$ may vary with time. If we set $t=Tt^{\prime }$, with T=1/g, then $V_{i}=U_{i}g$. Changing variables and dropping the ${}^{\prime }$ symbol to simplify, we find

(A3) $$\begin{array}{cc} \frac{V_{3}}{gh}\left[\phi v_{3,x_{3}}+\phi_{x_{3}}v_{3}\right]=\phi , & \frac{V_{3}}{h}v_{3,x_{3}}=\frac{P}{h^{2}\zeta }\left[p_{x_{3}x_{3}}{\left(1-\phi \right)}^{2}-2\left(1-\phi \right)\phi_{x_{3}}p_{x_{3}}\right], \end{array}$$

(A4) $$\begin{array}{cc} \frac{\mu_{s}U_{1}R_{0}}{Ph^{2}}u_{1,x_{3}x_{3}}=p_{x_{1}}+\frac{\Pi }{P}\phi_{x_{1}}, & \left(2\mu_{s}+\lambda_{s}\right)\frac{U_{3}}{Ph}u_{3,x_{3}x_{3}}=p_{x_{3}}+\frac{\Pi }{P}\phi_{x_{3}}, \end{array}$$

with boundary conditions:

(A5) $$\begin{array}{c} \begin{array}{c} \left(u_{1},u_{3}\right){|}_{x_{3}=0}=\left(0,0\right),\;u_{1,x_{3}}{|}_{x_{3}=1}=0,\;\left[-\left(\frac{\Pi }{P}\phi +p\right)+\left(2\mu_{s}+\lambda_{s}\right)\frac{U_{3}}{hP}u_{3,x_{3}}\right]{|}_{x_{3}=1}=-{\left(\frac{\Pi }{P}\phi +p\right)}_{ext}. \end{array} \end{array}$$

We can get an approximate solution of (A3) and (A4) in the same asymptotic limit as in [11] with slight variations due to the fact that we have an equation for the solid biomass displacements, not for fluid biomass velocities.

First, set $V_{3}=gh$ and $P=gh^{2}\zeta$, to balance growth and flow in (A3). For the rest, we argue with (A4). We set $K_{3}=\frac{\mu_{s}U_{1}R_{0}}{Ph^{2}}$, $K_{2}=\frac{\left(2\mu_{s}+\lambda_{s}\right)U_{3}}{Ph}=\frac{2\mu_{s}+\lambda_{s}}{\mu_{f}}\frac{\xi_{\infty }^{2}}{gh^{2}}$ and $\varepsilon_{p}=\frac{P}{\Pi }$, obtaining

(A6) $$\begin{array}{cc} (\phi_{x_{3}}v_{3}+\phi v_{3,x_{3}})=\phi , & v_{3,x_{3}}=p_{x_{3}x_{3}}\left(1-\phi^{2}\right)-2p_{x_{3}}\phi_{x_{3}}\left(1-\phi \right), \end{array}$$

(A7) $$\begin{array}{cc} K_{3}u_{1,x_{3}x_{3}}=p_{x_{1}}+\varepsilon_{p}^{-1}\phi_{x_{1}}, & K_{2}u_{3,x_{3}x_{3}}=p_{x_{3}}+\varepsilon_{p}^{-1}\phi_{x_{3}}, \end{array}$$

with $\left(u_{1},u_{3}\right){|}_{x_{3}=0}=\left(0,0\right),$$u_{1,x_{3}}{|}_{x_{3}=1}=0,$$\left[\left(-\varepsilon_{p}^{-1}\phi -p\right)+2K_{2}u_{3,x_{3}}\right]{|}_{x_{3}=1}={\left(-\varepsilon_{p}^{-1}\phi -p\right)}_{ext}.$ Next, we assume $\varepsilon_{p}<<1$ and expand in powers of $\varepsilon_{p}$: $a=a^{\left(0\right)}+\varepsilon_{p}a^{\left(1\right)}+O\left(\varepsilon_{p}^{2}\right)$ where $a=u_{1},u_{3},v_{1},v_{3},\phi ,p$. The equations for the displacements (A7) impose $\phi_{x_{1}}^{0}=\phi_{x_{3}}^{0}=0$. Thus, to leading order $\phi^{0}=\phi_{\infty }$ and $\phi =\phi_{\infty }+\varepsilon_{p}\phi^{\left(1\right)}+O\left(\varepsilon_{p}\right).$ Inserting the expansions in Equations (A6) and (A7) and equating coefficients to zeroth order ($\varepsilon_{p}^{0}$) we find

(A8) $$\begin{array}{c} v_{3,x_{3}}^{0}=1,\;v_{3,x_{3}}^{0}={\left(1-\phi_{\infty }\right)}^{2}p_{x_{3},x_{3}}^{\left(0\right)}, \end{array}$$

(A9) $$\begin{array}{c} K_{3}u_{1,x_{3}x_{3}}^{\left(0\right)}=p_{x_{1}}^{\left(0\right)}+\phi_{x_{1}}^{\left(1\right)},\;K_{2}u_{3,x_{3}x_{3}}^{\left(0\right)}=p_{x_{3}}^{\left(0\right)}+\phi_{x_{3}}^{\left(1\right)}, \end{array}$$

with boundary conditions $\left(u_{1}^{\left(0\right)},u_{3}^{\left(0\right)}\right){|}_{x_{3}=0}=\left(0,0\right),$ $u_{1,x_{3}}^{\left(0\right)}{|}_{x_{3}=1}=0,$ $\left(-p^{\left(0\right)}+2K_{2}u_{3,x_{3}}^{\left(0\right)}\right){|}_{x_{3}=1}=-p_{ext}.$ We have used $\phi_{ext}=0$ and included $\varepsilon_{p}^{-1}\phi_{\infty }$ in a term $\varepsilon_{p}^{-1}p^{\left(-1\right)}$, $\phi_{\infty }$ being constant the derivatives $p_{x_{1}},p_{x_{3}},p_{t}$ do not contain this term. Equation (A8) gives $v_{3}^{\left(0\right)}=x_{3}$. Integrating in time, we get $u_{3}^{\left(0\right)}=x_{3}t.$ Introducing this in Equation (A9) we obtain

(A10) $$\begin{array}{c} p_{x_{3}}^{\left(0\right)}=-\phi_{x_{3}}^{\left(1\right)}. \end{array}$$

Introducing (A10) in (A8) we find $1=-{\left(1-\phi_{\infty }\right)}^{2}\phi_{x_{3}x_{3}}^{\left(1\right)},$ with boundary conditions $\phi_{x_{3}}^{\left(1\right)}\left(1\right)=0$ and $\phi_{x_{3}}^{\left(1\right)}\left(0\right)=\phi^{\left(1\right)}\left(0\right)\frac{h}{\ell }$, where *ℓ* is the lengthscale over which gradients develop in the substrate due to water flowing to the biofilm, approximated by *R*. The latter boundary condition is established in [11] by matching a two phase flow model for the biofilm with another one for agar, we keep it here. The solution consistent with these boundary conditions is $\phi^{\left(1\right)}=\frac{1}{{\left(1-\phi_{\infty }\right)}^{2}}\left(x_{3}-\frac{x_{3}^{2}}{2}+\frac{R}{h}\right),$ which gives $p^{\left(0\right)}=p^{\left(0\right)}{|}_{x_{3}=1}+\left(\phi^{\left(1\right)}{|}_{x_{3}=1}-\phi^{\left(1\right)}\right)=p_{ext}+2K_{2}t+{\left(1-\phi_{\infty }\right)}^{-2}\left(\frac{x_{3}^{2}}{2}-x_{3}+\frac{1}{2}\right),$ where the contribution $\varepsilon^{-1}=\frac{R}{h}$ disappears thanks to the boundary condition. For the expansion of ϕ in powers of $\varepsilon_{p}$ to be consistent we need $\varepsilon_{p}/\varepsilon <<1$. Notice that $u_{3}^{\left(0\right)}=x_{3}t$ implies $u_{3,x_{3}}^{\left(0\right)}{|}_{x_{3}=1}=t$. To compute $u_{1}$ and $v_{1}$ we use Equation (A9), and the derivatives with respect to $x_{1}$ of *p* and ϕ, which enter this equation through the dependence on $h(x_{1},t).$ To address this issue properly, we switch back to dimensional variables using $P=gh^{2}\zeta$ and $x_{3}^{\prime }=x_{3}/h$ to get

(A11) $$\begin{array}{c} p=\frac{g\mu_{f}}{\xi_{\infty }^{2}{\left(1-\phi_{\infty }\right)}^{2}}\left(\frac{x_{3}^{2}+h^{2}}{2}-x_{3}h\right), \end{array}$$

(A12) $$\begin{array}{c} \phi =\phi_{\infty }+\varepsilon_{p}\phi^{\left(1\right)}=\phi_{\infty }+\frac{g\mu_{f}}{\Pi \xi_{\infty }^{2}{\left(1-\phi_{\infty }\right)}^{2}}\left(x_{3}h-\frac{x_{3}^{2}}{2}+Rh\right), \end{array}$$

discarding in *p* the lower order terms which do not contribute to the derivatives. Then, Equation (A9) yields $\mu_{s}u_{1,x_{3}x_{3}}=\frac{g\mu_{f}}{\xi_{\infty }^{2}{\left(1-\phi_{\infty }\right)}^{2}}hh_{x_{1}}\left(1+\frac{R}{h}\right).$ Integrating twice and applying the boundary conditions (A2) at 0 and *h* we find the displacement

(A13) $$\begin{array}{c} u_{1}=\frac{g\mu_{f}}{\xi_{\infty }^{2}\mu_{s}{\left(1-\phi_{\infty }\right)}^{2}}Rh_{x_{1}}\left(\frac{x_{3}^{2}}{2}-x_{3}h\right). \end{array}$$

Differentiating the displacements $u_{1}$ and $u_{3}$ with respect to time we get the velocities

(A14) $$\begin{array}{c} v_{1}=\frac{g\mu_{f}}{\xi_{\infty }^{2}\mu_{s}{\left(1-\phi_{\infty }\right)}^{2}}\left({\left[Rh_{x_{1}}\right]}_{t}\left[\frac{x_{3}^{2}}{2}-x_{3}h\right]-Rh_{x_{1}}x_{3}h_{t}\right),\;v_{3}=x_{3}. \end{array}$$

Once we approximate expressions for velocities, pressures, and volume fractions are available in the $x_{1}x_{3}$ plane, the equation for the free boundary (18) becomes $h_{t}+\frac{\partial }{\partial x_{1}}\int_{0}^{h}v\cdot {\hat{x}}_{1}\;dx_{3}=v\cdot {\hat{x}}_{3}{|}_{0},$$v=v_{s}-\frac{{\left(1-\phi \right)}^{2}}{\zeta }\nabla p,$ where v is the volume averaged velocity and $v_{s}$ the velocity of the solid, given by (A14). Setting $\phi ∼\phi_{\infty }$, we have $v\cdot {\hat{x}}_{3}{|}_{0}=gh\frac{{\left(1-\phi \right)}^{2}}{{\left(1-\phi_{\infty }\right)}^{2}}∼gh,$ and the flux becomes $\int_{0}^{h}v\cdot {\hat{x}}_{1}\;dx_{3}=\frac{g\mu_{f}}{\xi_{\infty }^{2}\mu_{s}{\left(1-\phi_{\infty }\right)}^{2}}\left(-{\left[Rh_{x_{1}}\right]}_{t}\frac{h^{3}}{3}-Rh_{x_{1}}\frac{h^{2}}{2}h_{t}\right)-g\frac{h^{2}h_{x_{1}}}{2}.$ Performing the change of variables $h=e^{t}\tilde{h}$, $h_{t}=e^{t}{\tilde{h}}_{t}+e^{t}\tilde{h}$, and dropping the symbol˜ for simplicity, we find the desired equation.

In radial coordinates the same arguments work. The equation for the height is then:

$$\begin{array}{c} h_{t}-\frac{g\mu_{f}}{\xi_{\infty }^{2}\mu_{s}{\left(1-\phi_{\infty }\right)}^{2}}\frac{1}{r}{\left(r({\left[Rh_{r}\right]}_{t}\frac{h^{3}}{3}+Rh_{r}\frac{h^{2}}{2}h_{t})\right)}_{r}-g\frac{1}{r}{\left(r\frac{h^{2}h_{r}}{2}\right)}_{r}=gh. \end{array}$$

In dimensionless variables $r=R_{0}\tilde{r}$, $t=g^{-1}\tilde{t}$, $h=h_{0}\tilde{h}$. Dropping the symbol ˜ for simplicity the equation becomes $h_{t}-K\frac{1}{r}{\left(r({\left[\frac{R}{R_{0}}h_{r}\right]}_{t}h^{3}+\frac{R}{R_{0}}h_{r}\frac{3h^{2}}{2}h_{t})\right)}_{r}-\frac{h_{0}^{2}}{2R_{0}^{2}}\frac{1}{r}{\left(rh^{2}h_{r}\right)}_{r}=h,$ with $K=\frac{g\mu_{f}}{3\xi_{\infty }^{2}\mu_{s}{\left(1-\phi_{\infty }\right)}^{2}R_{0}}h_{0}^{3}.$ Assuming $\epsilon =h_{0}/R_{0}<<K$ we get

(A15) $$\begin{array}{c} h_{t}-K\frac{R_{t}}{R_{0}}\frac{1}{r}{\left(rh_{r}h^{3}\right)}_{r}-K\frac{R}{R_{0}}\frac{1}{r}{\left(rh_{rt}h^{3}\right)}_{r}-K\frac{R}{R_{0}}\frac{1}{r}{\left(rh_{r}\frac{3h^{2}}{2}h_{t}\right)}_{r}=h. \end{array}$$

Performing the change of variables $h=e^{t}\tilde{h}$, $h_{t}=e^{t}{\tilde{h}}_{t}+e^{t}\tilde{h}$, and dropping again the symbol˜ for simplicity, we find (19).
